# A regulator of early flowering in barley (*Hordeum vulgare* L.)

**DOI:** 10.1371/journal.pone.0200722

**Published:** 2018-07-17

**Authors:** Ahmed Ibrahim, Matthew Harrison, Holger Meinke, Yun Fan, Peter Johnson, Meixue Zhou

**Affiliations:** 1 Tasmanian Institute of Agriculture, University of Tasmania, Tasmania, Australia; 2 Department of Plant Science, Institute for Agricultural Research, Ahmadu Bello University, Zaria, Nigeria; GERMANY

## Abstract

Heading date (HD) of cereals is an important trait for adaptation to diverse environments and is critical for determining yield and quality and the number of genes and gene combinations that confer earliness in barley under short days is limited. In our study, a QTL for early flowering was identified from the cross between an Australian malting barley cultivar and a Chinese landrace. Four sets of near isogenic lines (NILs) were developed with a QTL located on chromosome 5H at the interval of 122.0–129.0 cM. Further experiments were conducted to investigate how this gene was regulated by photoperiod using the NILs with three sowing dates from autumn to summer. The NILs carrying the earliness allele were significantly earlier than the late genotype at all sowing dates. This gene was different from previously reported vernalisation genes that are located at a similar position as no vernalisation was required for all the NILs. The difference between this gene and *Eam5* (*HvPHYC*) locus which also located between two co-segregated markers (3398516S5, 122.5 cM, and 4014046D5, 126.1 cM), is that with the existence of *Ppd-H1* (*Eam1*), *Eam5* has no effect on ear emergence under long days while the gene from TX9425 still reduced the time to ear emergency. The locus showed no pleiotropic effects on grain pasting properties and agronomic traits except for spike length and number of spikelets per spike, and thus can be effectively used in breeding programs. The array of early heading dates caused by interactions of Eam5 gene with other maturity genes provides an opportunity to better fine tune heading dates with production environments, which can be critical factor in barley breeding.

## Introduction

Barley is an important cereal crop grown worldwide under a wide range of environments [1]. The broad adaptation of this crop to varying climatic and regional conditions is in part caused by the diversity in flowering time (anthesis, Zadoks GS61) or heading date (HD, Zadoks GS51) or tipping (awn emergence, Zadoks GS 49) [2–4]. These terms are often used interchangeably by many scientist [4]. The main factors affecting HD are photoperiod, vernalization, temperature and management [5–10]. These factors provide the physiological and genetic basis for variations in the duration of developmental stages, such as double ridge (DR), terminal spikelet (TS), heading, anthesis and grain filling [11, 12]. Genotypes vary in their photoperiodic response [13, 14], with temperature being very important to plant physiological processes [15] especially variations in duration to spikelet initiation [16], heading and flowering (anthesis) in cereals [14, 17]. It follows that a linear association generally describes the relationship between cumulative temperatures over the growing season and heading/anthesis in barley [16, 18, 19] and in many other cereals. To initiate flowering, winter barley requires vernalisation, i.e. exposure to prolonged temperatures below 10°C for a period between 4 to 6 weeks [20, 21, 22].

Early maturity of cool-season cereals like barley under short-day environments is vital in many grain producing regions of the world. Barley grown in Eastern Asia has evolved unique earliness mechanisms that stimulate early ear emergence under short days even when vernalization is not necessary. These mechanisms may involve several maturity genes interacting together in either additive, epistatic or pleiotropic way in order to regulate earliness. Apart from these allelic and no-allelic interactions, intra-locus mutations at *Vrn* loci, *Eps* and *HvPHYC* loci are responsible for earliness in both barley and wheat [23, 24] with a rich allelic variation at *Vrn-H1* or/and *HvPhyC* [25]. Our understanding of these interactions is gradually improving as we learn more about their mechanisms of expression and that some of these genes are functional under long days. Four major genes are reported to be responsible for vernalization. These include *Vrn-H1* (*Sgh2*), *Vrn-H2* (*Sgh1*), *Vrn-H3*(*Sgh3*) and *Vrn4* on chromosomes 5H, 4H, 7H and 5H, respectively [21, 26–31]. These four loci interact in an epistatic fashion to determine vernalization sensitivity in barley and wheat [32]. For example, the winter barley cultivars which are responsive to vernalization have *vrn-H1*_*Vrn-H2*_*vrn-H3* haplotype [29, 32, 33] and more recently were found to have the recessive mutant *vrn4* [34]. A model of heading-time regulation in both photoperiod groups (*Ppd-H1*; *ppd-H1*) under long day condition was proposed by Alqudah et al [25].

Heading date in barley is regulated by photoperiod response genes; the first identified being *Ppd-H1* (*Eam1*), which is a pseudo-response regulator gene (*HvPRR37*) that is effective under long days and is located on chromosome 2H [25, 35–37]. The second photoperiod gene, *Ppd-H2* (*HvFT3*), is located on chromosome 1H and regulates flowering time under short days [38, 39].

Earliness in intrinsic or per se genes determine the time and duration of reproductive phases [40, 41]. These QTL manifest their expression after all sources of variation in basic vegetative period (BVP) or maturity-related traits (such as vernalization and photoperiod) have been met [42–46]. Thus, Eps QTL are important in fine-tuning HD and anthesis in barley [41, 47, 48]. Eps QTL have significant effects on the time and duration of reproductive phase and spikelet number [41] which directly affect the grain yield [48]. Eps QTL also have significant effects on grain protein [49] which is inversely related to the starch [50, 51]. The *EARLY FLOWERING3* (*ELF3*) locus regulates flowering under the influence of photoperiod [52]. The recessive allele (*elf3*, *eam8*, *mat-a)* of this gene in barley causes early flowering in both short days (SDs) and long days (LDs) [52] and an insensitivity of barley to photoperiod [35, 52].

Apart from the Eps QTL, an early maturity factor in chromosome 5H was reported by Wexelsen [53]. The gene belongs to the family of photoreceptors, phytochromes, which helps plants to perceive, interpret, and translate light signals that modulate and synchronize their growth and development in any given environments [54, 55]. These phytochromes are involved in plant metabolism including flowering, shade avoidance, dormancy, and germination [56]. Close linkage to the rough awn trait demonstrates that *Eam5* is likely the locus reported by Laurie et al. [28] and later identified at the *HvPHYC* locus after which Szucs et al. [57] mapped to a similar position as that of *Vrn-H1*. *HvPHYTOCHROME C* (*HvPHYC*) is reported to be a candidate gene to *Eam5* [58]. Nishida et al. [59] found that a mutation at *HvPHYC*, due amino-acid substitution in the GAF domain, influenced HD under LDs. It was later found to affect ear emergence under long and non-inductive short days [58, 60]. Thus, *Eam5* likely interacts with many photoperiod response loci under an array of photoperiods. In addition, a casein kinase alpha (*HvCK2α-5H*) gene is also reported to be closely linked to the *Vrn-H1* [59]. This gene encodes the α subunit of CK2 protein and regulates flowering, which is found in cereals including rice, wheat and barley under both short and long days [59].

Beside the photoperiod and vernalization genes which have been cloned, there is little information on the genetics, physiological and biochemical functions of other genes regulating early ear emergence in barley. For instance, *HvPHYC* gene has been recently fine mapped but there is discrepancy surrounding the expression and interaction of this gene with the environment. Further work is required to determine their quantitative effects [61]. This manuscript provides some details about interactions of one component of the circadian clock with various photoperiod regimes in essentially fixed genetic background in barley.

From thousands of F_10_ recombinant inbred lines from the cross of TX9425 and Franklin, we have identified another early flowering allele that may be distinct from other QTL on chromosomes 2H, 3H and 6H which were reported earlier [39]. Four pairs of near isogenic lines (NILs) were developed to investigate: 1) the location of this early flowering gene; and 2) the effect of this gene on agronomic traits, yield components, and grain quality.

## Materials and methods

### Development of near-isogenic lines

**Fig 1:**
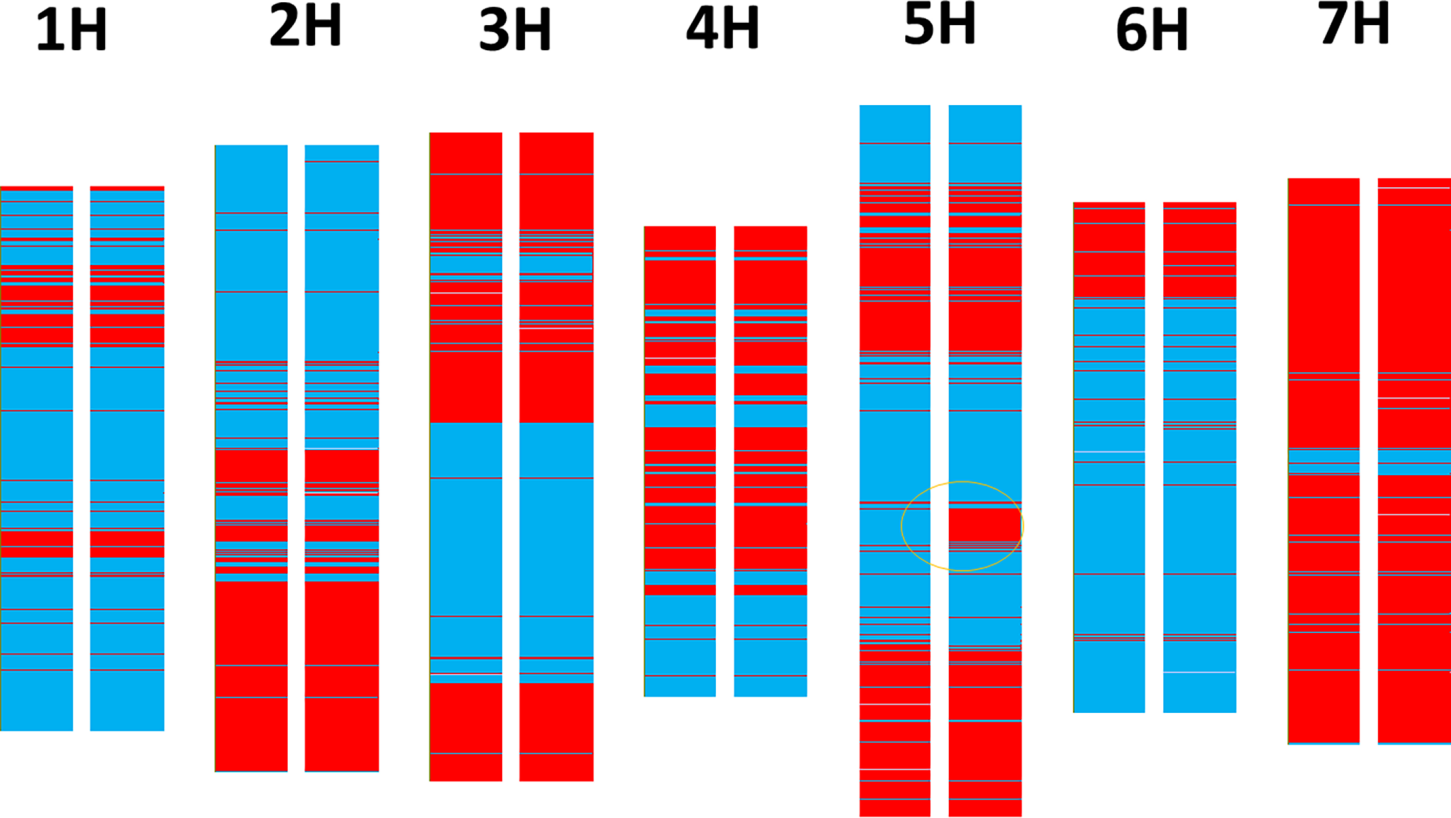
Comparison between genotypes of near isogenic lines (Eps5HL-317-1-E, left, vs Eps5HL-317-1-L, right) from the cross of TX9425/Franklin. Red: Franklin genotype; blue: TX9425 genotype. The major difference is on 5H at the position of 122 to 129 cM (circled).

NILs were selected from over 1,000 F_9_ derived F_10_ recombinant inbred lines (RILs) of the cross of TX9425 and Franklin. Franklin (Shannon/Triumph) is a late maturing malting variety from Australia [62] with the seeds being sourced from the University of Tasmania and the Australian Grains Genebank. TX9425 (Taixing 9425) is a Chinese landrace which has semidwarf and early maturity genes, most likely the dominant alleles of the *Ppd-H1*, the spring *Vrn-H1* allele and the recessive allele of the *Eps5HL* [39]. From the RIL population four lines segregating on heading date were further selfed to produce homozygous early and late lines, leading to four pairs of the NILs. They are Eps5HL-116 (-E/-L), Eps5HL-317-1(-E/-L), Eps5HL-317-2 (-E/-L), Eps5HL-322 (-E/-L) with–E carrying earliness allele. All NILs have both the spring *Vrn-H1* allele as vernalization is not needed to all lines and *Ppd-H1* since the segment containing this gene is from TX9425 (Fig 1).

### Genotyping the NILs

Genomic DNA of NILs was extracted from the leaf tissue of four-week old seedlings, according to the plant DNA extraction protocol for DArT analysis (https://www.diversityarrays.com/files/DArT_DNA_isolation.pdf). The two parental cultivars and four pairs of NILs were genotyped with DArTseq (http://www.diversityarrays.com/dart-application-dartseq). Around 10,000 polymorphism molecular markers with known positions were chosen for comparing the differences between NILs and the relationships to their parents.

### Field experimentation

Field experiments were conducted at Mt Pleasant Laboratories, at the Tasmanian Institute of Agriculture (TIA) in Launceston, Tasmania (Latitude: -41.4702 Longitude: 147.1392), where the day length ranges from 9 to 15 hours (S1 Fig). The four pairs of NILs and both parents were sown in tanks (1.50 x 3.0 x 1.0 m) with a spacing of 20.0 cm x 7.0 cm (S2 Fig). Tanks were filled with sandy loam soils and fitted with irrigation facilities to avoid any water stress. The NILs/parents were grown in a randomized complete block design with three replications. Three different sowing dates, 14/January/2015 (SD1, summer), 13/March/2015 (SD2, summer/autumn) and 15/May/2015 (SD3, autumn), were selected to represent the photoperiods/temperatures (S1 Fig). Agronomic practices such as fertilizer rate and regular weeding were similar to local practices. Traits measured shown in Table 1 include: heading date (HD) (when the first spikelet emerged in one of the tillers of 50% of the plants) in calendar time, growing degree days to heading date (GDD), plant height (PH), number of nodes on the main plant, peduncle length, spike length, awn length and number of fertile spikelets per spike. Harvested grain was tested for pasting properties. Climatic data were taken from the nearest meteorological station, and GDD accumulation was calculated following the classical method:

$$GDD=\sum_{i=1}^{n}\left[\left(\frac{T_{\min}+T_{\max}}{2}-T_{b}\right)\right]$$

Where *n* = number of days taken for a particular growth stage to be accumulated, and *T*_min_, *T*_max_ = minimum and maximum daily air temperatures in ^o^C, respectively, and T_b_ = base temperature threshold for barley, which is 0 ^o^C [63]

**Table 1:** Traits scored and their abbreviations.

Traits	Symbol	Unit	Description	
Heading date	HD	d	Number of days from sowing to when fifty percent of the spike appears	
Thermal time (growing degree days)	TT/GDD	^o^Cd	Accumulated thermal time from day 0 of the sowing day to current growth stage	
Plant height	PH	cm	Measured from collar to the peak of the awns	
Spike length	SpkL	cm	Measured from the base of the spike to the tip	
Spikelet number	SpkN	Spk/spike	Number of spikelets in a spike	
Peduncle length	PedL	cm	Measured from the last node to the base of the spike	
Rapid Visco-analyser Unit	RVU	RVU	An RVU is approximately equal to 10 cP.	
Peak Viscosity	PV	RVU	Highest viscosity during cooking	
Time To Peak Viscosity	TTPV	min	Time taken to reach the peak	
Trough	TR	RVU	Lowest viscosity after cooling started	
Breakdown	BD	RVU	Peak viscosity minus trough (PV-TR)	
Final Viscosity	FV	RVU	Maximum viscosity after the temperature had returned to 50 ^o^C	
Setback	SB	RVU	Final viscosity minus trough (FV-TR)	
Pasting Temperature	PT	^o^C	Temperature when the rate of increase in viscosity reaches 11.5 RVU in 0.2 min	

### Analysis of pasting properties

The determination of pasting properties was conducted according to the methods described by [64, 65]. After harvesting and threshing, the samples in each NIL pair were air dried. 10.0 g of grains were sampled from each of the NIL pairs in each replication and ground in a Cylotech 1903 Mill. 4.0 g of the flour was slurried into 25.0 g of 0.1M of silver nitrate (AgNO_3_) solution in an aluminium canister. The slurry was then thoroughly mixed by moving the paddle both vertically and stirring in the canister before placing it into a Rapid Visco-Analyser (RVA-4D, Newport Scientific, Australia). The RVA instrument was used to determine the pasting properties. The RVA instrument was used for 10 s at 960 rpm then reduced to 160 rpm for the remainder of the test run. The initial temperature was 50 ^o^C, held for 1.0 minute, then heated to 95 ^o^C for 3.7 minutes and was maintained at 95 ^o^C for 2.5 minutes before cooling to 50 ^o^C over 3.8 min, and finally maintained at 50 ^o^C for 2.0 min. The measured parameters for pasting properties include: peak viscosity (PV), highest viscosity during heating; time to peak viscosity (TTPV); trough (T), lowest viscosity after cooling started; breakdown (BD), PV minus T; final viscosity (FV), highest viscosity after the temperature had returned to 50 ^o^C; setback (SB), FV minus T; pasting temperature (PT), temperature at which the trace left the baseline [65].

### Statistical analysis

SAS version 9.4 was used to conduct ANOVA to estimate the significances of the differences between each of the pairs, whilst the mean of each trait within genotypes was ranked used Tukey’s test [66].

## Results

### Mapping early flowering QTL

**Fig 2:**
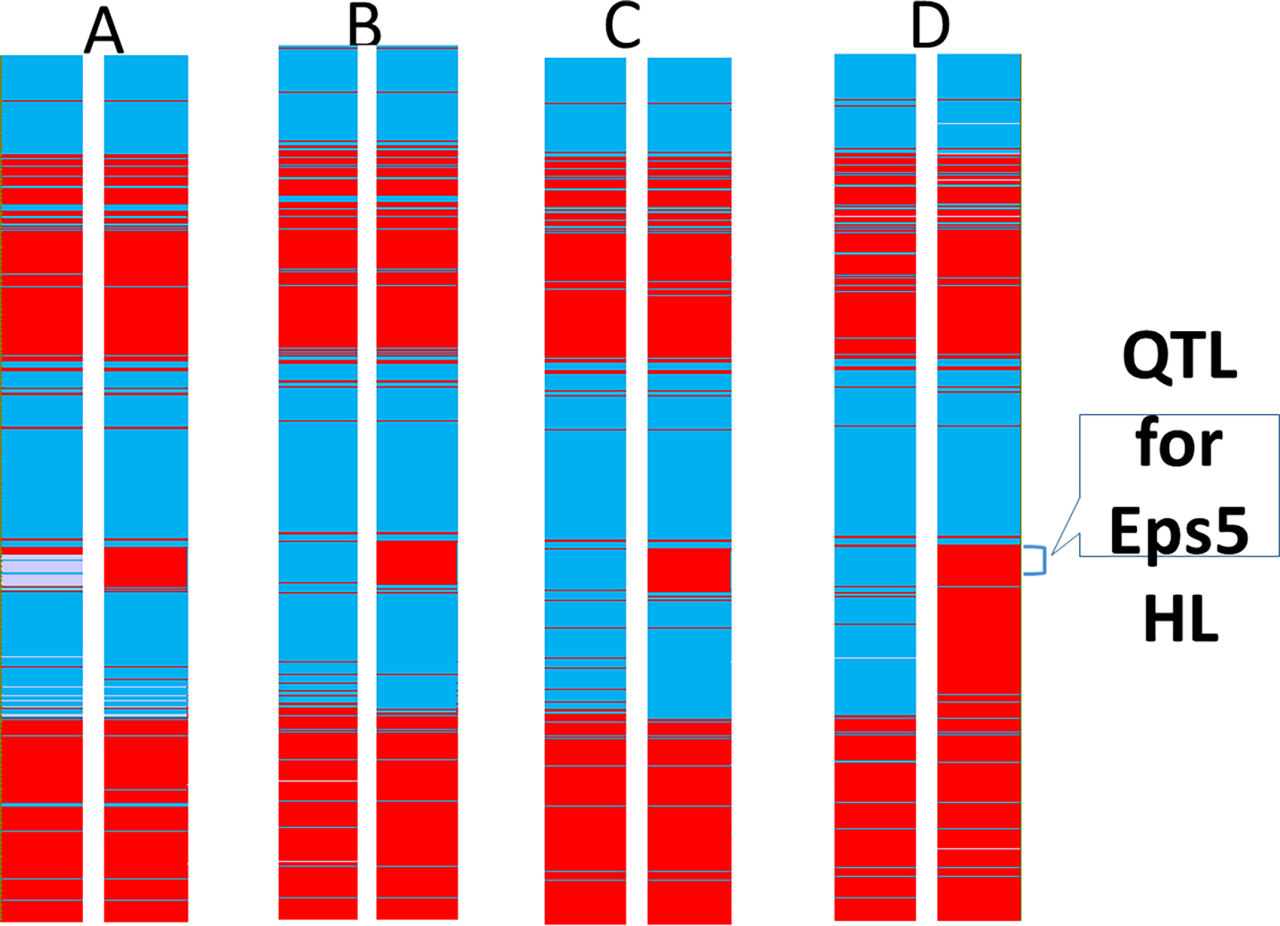
Comparison of genotypes of 5H for different pairs of near isogenic lines from the cross of TX9425/Franklin. A) Eps5HL-316-E/L; B) Eps5HL-317-1-E/L; C) Eps5HL-317-2-E/L; and D) Eps5HL-322-E/L. Red: Franklin genotype; blue: TX9425 genotype; white: heterozygous.

The genotyping of the four pairs of NILs was conducted using DArTseq with over 30,000 markers. Fig 1 shows that for the Eps5HL-317-1 pair, except for the 122–129 cM region of chromosome 5H, genetic background was identical for the early (Eps5HL-317-1-E) and late (Eps5HL-317-1-L) lines. Similar regions were located in the other three pairs of NILs except that the region with different background was much greater (122–140 cM) (Fig 2) for the Eps5HL-322 pair. The earliness allele was from TX9425. More than 100 SNP/DArT markers co-segregated with the trait. As reported previously [39] two major QTL for early maturity were found in a DH population originating from the cross of TX9425 and Franklin. One QTL is located on chromosome 3H that is closely linked to *sdw1* and *uzu1* genes. This early maturity allele is absent in all these NILs. The other early maturity QTL is located on chromosome 2H (most likely *Eam1*) which exits in all the NILs (data not shown).

### Effects of sowing time on heading dates (HD) and GDD

**Fig 3:**
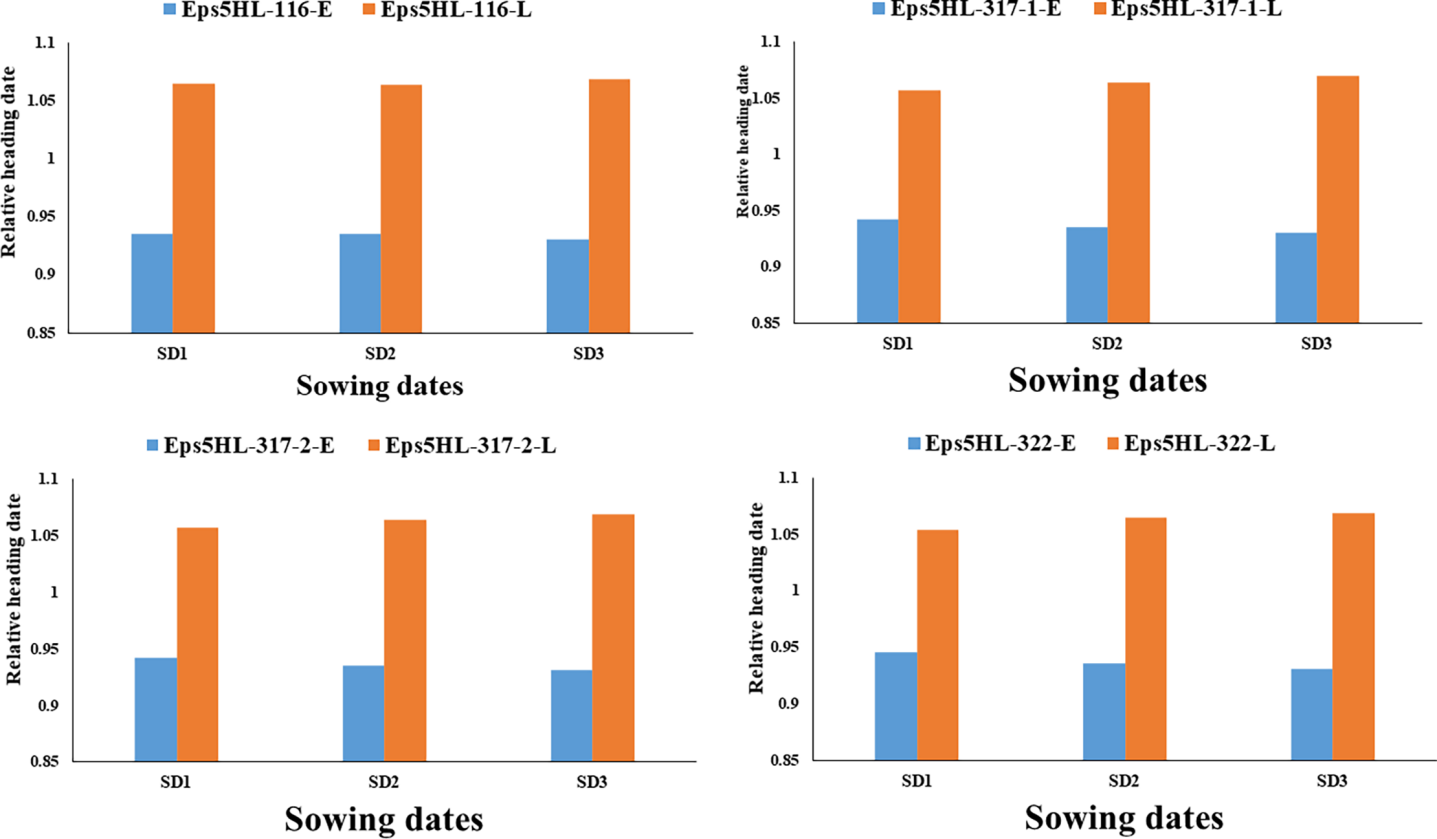
Relative heading date of different pairs of NILs, TX9425 and Franklin from different sowing dates.

Sowing dates resulted in significant differences in HD and GDD (Table 2, S3 Fig). All four NILs and their parents (TX9425 and Franklin) had the fewest days to heading in SD1, followed by SD2 and SD3 (Table 2). Consistent differences between lines with early and late alleles existed in all the sowing dates. Heading days (HDs) for TX9425 were 41, 105 and 125 d for SD1, SD2 and SD3, respectively. HDs for Franklin were 55, 151 and 162 for SD1, SD2 and SD3, respectively. All NILs were earlier than Franklin but later than TX9425 except SD1 with the early genotypes of the NILs and TX9425 flowering at same time. HDs of the NILs with the early allele were 41, 131 and 136 for SD1, SD2 and SD3, respectively, while those with the late allele were 45, 149 and 155 for SD1, SD2 and SD3, respectively (Table 2). The NILs carrying the early allele were approximately four days earlier than those with the late alleles in SD1. The differences between the two alleles were much greater with 18 and 20 days in SD2 and SD3, respectively (Table 2). The relative HDs for each sowing date were similar for all pairs across sowing dates, with less difference in SD1 (Fig 3). When considering the effects of accumulated temperature for the HD to be expressed, similar trend was observed in the GDD with consistent differences among the SDs and among the genotypes except in Franklin (S2 Fig)

**Table 2:** Means of heading dates and other different traits of two parent varieties and four pairs of NILs under different sowing dates*.

Genotype		HD	GDD	PH	SpkL	SpkN		PedL	InterL
				**SD1**					
TX9425		41.0±2b	756.3±38.2b	36.0±0.7b	4.9±0.1b	20.7±1.5b		38.3±4.6a	16.6±0.8a
Franklin		55.0±4a	980.1±25.7a	47.1±2.6a	9.3±0a	24.3±1.2a		29.0±2b	16.8±2.5a
Eps5HL-116-E		42.3±1b	774.1±37b	87.9±0.5a	7.5±0.2b	22.0±1.2b		33.3±1.8a	17.6±0.8a
Eps5HL-116-L		46.7±1.3a	846.4±32a	87.7±0.8a	9.1±0.1a	25.0±1.0a		35.3±5a	15.0±1a
Eps5HL-317-1-E		41.1±1b	756.6±37.1b	83.9±0.2b	7.8±0.1b	21.3±1.5b		28.7±0.3a	19.3±1.7a
Eps5HL-317-1-L		46.0±1.7a	840.5±26.5a	95.9±4a	9.2±0.2a	25.3±1.5a		30.1±0.8a	17.7±0.7a
Eps5HL-317-2-E		41.2±2b	756.8±38b	84.2±1b	7.6±0.1b	22.4±1.5b		37.0±2.6a	19.0±1.3a
Eps5HL-317-2-L		45.3±1.6a	824±21.4a	89.7±2.5a	8.9±0.1a	25.0±1a		33.0±2.3b	16.2±1.7a
Eps5HL-322-E		41.0±1b	756.3±27b	70.0±1.1b	7.5±0.1b	21.2±2b		29.2±2a	20.2±2a
Eps5HL-322-L		45.7±1.1a	839.6±21a	76.5±1.3a	9.0±0a	25.0±1.7a		31.7±1.2a	15.6±2.5b
				**SD2**					
TX9425		105.0±5b	1065.2±42b	94.5±2.1a	6.0±0.3b	28.0±1.5b		25.7±2.2a	12.1±2.3b
Franklin		152.0±2.1a	1378.9±61a	84.7±7.1b	11.8±0.2a	29.0±1.1a		24.0±1.4a	14.2±2.1a
Eps5HL-116-E		131.0±2b	1238.1±23b	89.8±4.1a	10.0±0b	24.3±1.5b		30.4±2.3a	18.2±a
Eps5HL-116-L		148.9±3a	1351.0±14a	90.0±4a	11.8±0.2a	28.7±1.5a		33.4±2.3a	20.0±2.5a
Eps5HL-317-1-E		132.2±2b	1246.9±22b	97.1±8.6a	9.8±0.2b	25.3±0.5b		25.8±5a	14.2±2.2b
Eps5HL-317-1-L		149.0±3.1a	1351.6±27a	98.2±6.5a	12.0±0.2a	28.7±1.1a		24.1±9a	20.0±2.5a
Eps5HL-317-2-E		131.3±3b	1238.7±21b	82.1±2.8b	10.0±0b	24.3±0.6b		29.3±2a	20.0±2.2a
Eps5HL-317-2-L		149.0±2a	1351.6±25a	94.2±3.1a	11.8±0.3a	28.0±1.7a		29.1±2a	15.2±2.4a
Eps5HL-322-E		131.0±2b	1238.1±17b	89.4±3.3b	10.0±0b	24.3±0.6b		28.0±1.8a	18.0±1.4a
Eps5HL-322-L		149.4±2.6a	1352.0±18a	92.3±1.6a	11.8±0.3a	28.0±2a		26.8±1.3a	16.0±0.8a
				**SD3**					
TX9425		125.3±2.5b	925.2±28b	105.0±2.5a	6.8±0.2b	33.7±1.6b		35.0±3a	14.0±0.6a
Franklin		162.6±2.7a	1382.2±26a	86.7±3.6b	14.0±0.2a	39.3±1.5a		23.5±2b	16.0±0.2a
Eps5HL-116-E		135.0±2.6b	1029.6±24b	110.5±0.4a	11.4±0.2b	36.7±1.5b		41.0±3a	21.0±0.8a
Eps5HL-116-L		155.0±3a	1294.5±22a	110.2±0.5a	13.8±0.3a	38.3±1.4a		46.0±2a	22.0±0.7a
Eps5HL-317-1-E		134.9±2b	1029.0±21b	104.0±0.9b	11.2±0.1b	35.0±2b		36.0±1.7a	16.0±1.5b
Eps5HL-317-1-L		155.3±3a	1294.8±21a	115.0±2a	14.4±0.3a	38.7±1.6a		39.5±1.6a	22.0±2.6a
Eps5HL-317-2-E		136.1±3b	1041.8±28b	105.0±.09b	11.0±0.2b	35.3±2.5b		36.0±1.3a	17.0±1.5a
Eps5HL-317-2-L		155.1±3a	1294.6±24a	111.7±4.5a	13.9±0.2a	37.7±1.5a		37.5±1.3a	17.5±5a
Eps5HL-322-E		135.0±2.6b	1029.6±27b	98.8±0.8b	11.00.1b	33.7±2b		35.6±3b	16.0±1.4a
Eps5HL-322-L		155.1±3a	1294.6±22a	105.0±3.6a	15.3±0.2a	38.3±1.6a		42.5a	17.5±0.3a

*Means in same column for each NIL pair followed by same letter are not significantly different (P = 0.05): Means ±Standard deviation; Abbreviations are defined in Table 1.

### Effects of early heading on agronomic traits and yield components

Different heading dates were associated with some of the agronomic traits and yield components. The average spike length of genotypes carrying the late allele was 1.3 cm longer than the spike length of those carrying the early allele (Table 2). Similar results were obtained for the number of fertile spikelets per spike with SD3 having the most spikelets per spike. Genotypes carrying the late allele had more spikelets per spike that those carrying the early allele (Table 2).

No significant differences between early and late lines were observed for peduncle length and internode length for most of the NILs (Table 2 and S1 Table). Franklin had significantly shorter peduncle length (PedL) than NILs and TX9425 in sowing dates 1 and 3, although 317-2-L under sowing date 1 and 322-L under sowing date 3 also had shorter PedL. However, significant differences in plant height between early and late lines were found in three of the four pairs, with genotypes having the late allele being slightly taller than those having the early allele (Table 2).

Sowing dates had significant effects on overall performance of all the traits. Spike length was shorter in SD1 (7.5–9.2 cm) than that in SD2 (9.8–12.0 cm) and SD3 (11.1–14.3 cm). Similar results were found for other traits with those from SD1 being shorter and having fewer spikelets per spike (Table 2). The differences between the early and late alleles were consistent for different SDs even though significant interactions were observed between the alleles and spike length and spikelets number per spike.

### Effects of heading date on pasting properties

**Fig 4:**
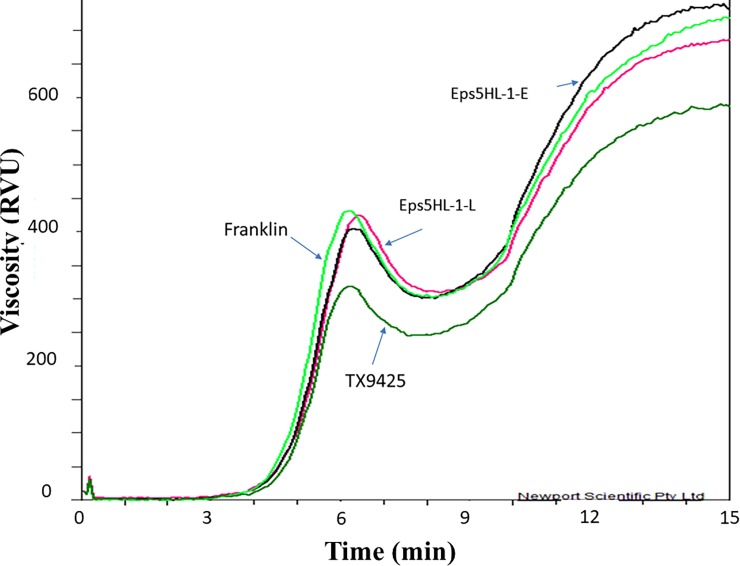
Effect of the Eps5HL locus on the starch pasting properties.

Fig 4 and Table 3 show that Franklin had generally higher PV, T, FV but lower BD, SB and PT values than TX9425. Both parents showed similar TTPV values. Differences were found between NIL pairs but no significant differences between early and late alleles were observed for all pasting properties (S2 Table). The Eps5HL-116, Eps5HL-317-1 and Eps5HL-317-2 pairs were very close to Franklin in most of the parameters while the Eps5HL-322 pair was close to TX9425 (Table 3). The Eps5HL-116 pair showed longer TTPV than both parents and other NIL pairs.

**Table 3:** Pasting properties of two parent varieties and four pairs of NILs*.

Variety/line	PV	T	BD	FV	SB	TTPV	PT
TX9425	412b	310b	102a	699b	389a	6.38a	84.2a
Franklin	451a	357a	94b	715a	358b	6.38a	82.1a
Eps5HL-116-E	450a	350a	100a	731a	381a	6.51a	81.0a
Eps5HL-116-L	448a	354a	93a	720a	365a	6.56a	79.3a
Eps5HL-317-1-E	454a	345a	110a	748a	403a	6.28a	79.7a
Eps5HL-317-1-L	435a	320a	115a	695a	375a	6.24a	80.8a
Eps5HL-317-2-E	444a	321a	123a	727a	406a	6.18a	80.7a
Eps5HL-317-2-L	467a	346a	122a	761a	415a	6.27a	78.4a
Eps5HL-322-E	417a	309a	109a	701a	392a	6.27a	82.2a
Eps5HL-322-L	427a	315a	111a	677a	362a	6.42a	83.9a

*Values in same column followed by same letter are not significantly different (P = 0.05). Comparisons are made within each pairs. Abbreviations are defined in Table 1.

## Discussion

### A heading date locus was identified in the long arm of chromosome 5H

TX9425, a Chinese landrace, has been reported to have different stress tolerances [67–73], semidwarf and early maturity genes [39]. In this study, a different gene/QTL was identified for heading date with the earliness allele derived from this cultivar. This locus was mapped to a position of 122–129 cM region of chromosome 5H using four pairs of near isogenic lines. The heading dates of the lines with the earliness allele occurred about 20 d earlier than those with the lateness allele in the normal growing season (autumn sowing) in Tasmania. This QTL was not identified in the DH population originating from the cross of the same parents (TX9425 and Franklin), most likely due to the relatively smaller effects which are often masked by the effects of two other major QTL [39] thus making difficult for comparative analysis with other major genes. By comparing the position in physical maps [74], the locus is situated at a similar position to *Vrn-H1*, a vernalisation gene [2] and two other flowering regulators, *HvPHYC* and *HvCK2α-5H* [58, 59].

*Vrn-H1* is expressed when plants are exposed to prolonged cold temperatures (vernalization) usually in winter cultivars to switch to reproductive phase [75]. Since TX9425 originated from East Asia and shows a spring growth habit, it likely has the spring *Vrn-H1* allele at this locus. Pankin et al. [58] reported that *Vrn-H1* locus is tightly linked to *HvPHYC* (*Eam5*) with no recombinants being detected between *Vrn-H1* and *HvPHYC* from a large BC_1_F_2:3_ population. Most of East Asian accessions have *HvPHYC* haplotypes 1, 3 and 4. These haplotypes existed in both winter and spring types even though the growth habit of most of these accessions were not defined [58]. Thus the *HvPHYC* haplotype in TX9425 needs to be further investigated.

The first report of an early maturity factor in chromosome 5H was by Wexelsen (1934) [53]. Close linkage to the rough awn trait demonstrates that this likely the locus reported by Laurie et al. (1995) [28] and later identified at the *HvPHYC* locus (*Eam5*). *Eam5* was mapped to the similar position of *Vrn-H1* [57] and *HvPHYC* is reported as the candidate gene [58–60]. This gene is well adapted to the environments of China and Japan and is found in ICARDA/CIMMYT genetic stocks (CMB85533, Higuerilla*2/Gobernadora) [58]. The amino-acid substitution in the GAF domain is the major influence of heading date under LDs [59]. However, *HvPHYC* may interact with other genes such as *Vrn-H1*, *sdw1* and *Ppd-H1* to induce early flowering under long and non-inductive short days [58, 60]. The QTL identified in this study behaved more like *HvPHYC* reported by Pankin et al. (2014) [58] who observed 23 and 3 days differences in flowering between the Bowman (late genotype) and Bowman(*eam5*) (early genotypes) under both short and long days, respectively. However, with the existence of *Ppd-H1*, *eam5* showed no significant effect on maturity, i.e. no differences between Bowman (*Ppd-H1*) and Bowman (*Ppd-H1* + *eam5*) while the QTL identified in this study still showed earliness, indicating a possible new allele for early flowering.

### Early heading affects some agronomic traits but not flour pasting properties

Previously reported eps QTL have been found to have direct influence on spike morphology, including length of the spike, spike density and thousand kernel weights in cereals [44, 76–78] since the duration of vegetative period is positively correlated to spike length and spikelet number per spike [79]. NILs carrying the late alleles including the late parent Franklin were found to have longer spike length and higher grain number than those carrying the early alleles across all sowing dates (Table 2), due to prolonged duration of the spike developmental stages [41, 76, 78].

*Ppd-H1* was reported to have pleiotropic effects on plant height [80] and a QTL regulating heading date was found to be closely linked to the *sdw1 (denso*) gene in barley [81]. *Ppd-H1* also seems to be one of the key genetic determinants for plant height and tiller number with *Ppd-H1* reducing the number of tillers per plant [82]. QTL regulating plant height in the DH population of TX9425 and Franklin are located on 2H and 3H [39], likely *uzu1* and *sdw1* (*denso*), respectively. However, none of these are in similar positions to early heading gene or are likely present in the NILs. Among four NIL pairs, 116 pair showed no significant difference in plant height, indicating no pleiotropic effect between the 5HL segment and plant height. The difference in the height between the alleles in the 317–1, 317–2 and 322 NIL pairs could be due to heading date or a different QTL responsible for plant height. Further studies are needed to confirm this.

Flour pasting properties are important quality traits and have close relationship with malting quality [83, 64] and food processing quality [65]. Pasting properties have been found to be influenced by genotype [83, 84] and environment [65, 84–86]. Earliness per se can also influence grain protein content [49] thus pasting properties [87]. Several QTL have been identified for pasting properties in barley [86]. These QTL for pasting properties are located on chromosomes 1H, 2H, 3H, 4H, 6H and 7H [86] with no QTL on 5H, indicating that may be unlikely that this chromosome segment would affect pasting properties. Indeed, this was confirmed in the current experiment, with no significant differences measured between NILs with the early and the late alleles.

In conclusion, a QTL on chromosome 5HL that causes variations in heading/flowering date and growing degree-days to heading was identified from the cross between TX9425 and Franklin. Using different pairs of NILs, the gene was mapped to 122–129 cM with a large number of co-segregating markers. This locus was found to have less sensitivity to temperature and photoperiod compared with other maturity/vernalisation genes at similar positions, indicating a possible new allele for early flowering. The chromosome region results in significant effects on some agronomic traits such as the length of spike and the number of spikelets per spike, but has less effect on flour pasting properties. Since the maturity effects of *Eam5* are highly variable, closely linked molecular markers could be useful in facilitating the utilization of this gene. These markers are much closer than previously reported Raw1 locus, which is about 5 cm away from this earliness locus.

## Supporting information

S1 Fig Mean monthly temperatures and day length hours per month in 2015 for Tasmania. Arrows are three different sowing dates.
(TIF)

S2 Fig Morphological differences in ear emergence, maturity and spike length of four pairs of NILs carrying Eps5-317-1-E and Eps5HL-317-1-L alleles. (TIF)

S3 Fig Growing degree-day to heading date of different pairs of NILs, TX9425 and Franklin from different sowing dates. (TIF)

S1 Table Mean square values from the analysis of variance for all the traits studied for each pair of the NILs (early and late) and the parents (TX9425 and Franklin). (DOCX)

S2 Table Mean square values from the analysis of variance for all the pasting properties studied for each pair of the NILs (early and late) and the parents (TX9425 and Franklin). (DOCX)
