# Supplementary figures and images for: A regulator of early flowering in barley (Hordeum vulgare L.)
Source: PLoS One. 2018 Jul 17;13(7):e0200722. doi: 10.1371/journal.pone.0200722 (PMC6049932; doi:10.1371/journal.pone.0200722)

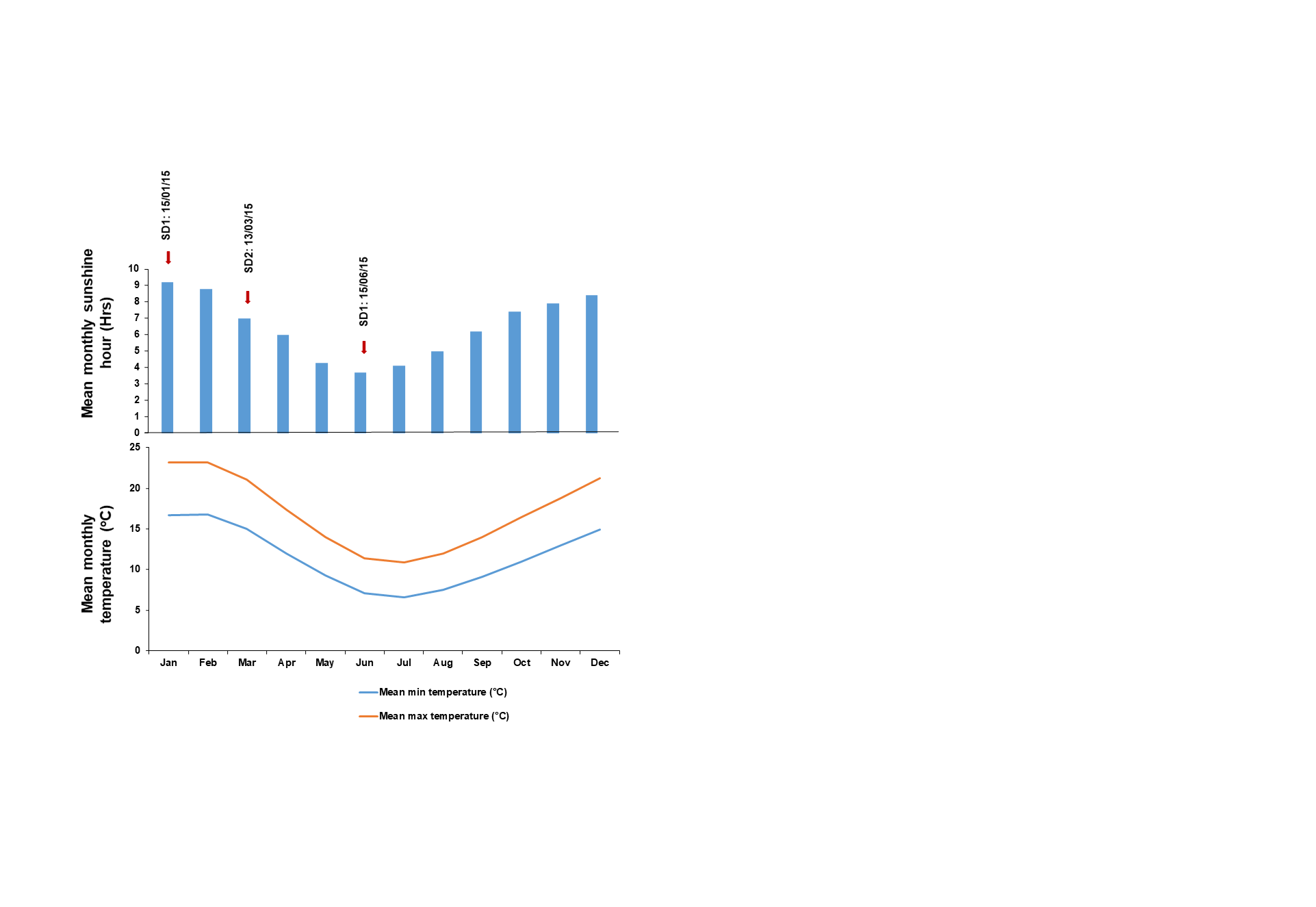

Supplement: S1 Fig — Arrows are three different sowing dates. (TIF) [file pone.0200722.s001.TIF]

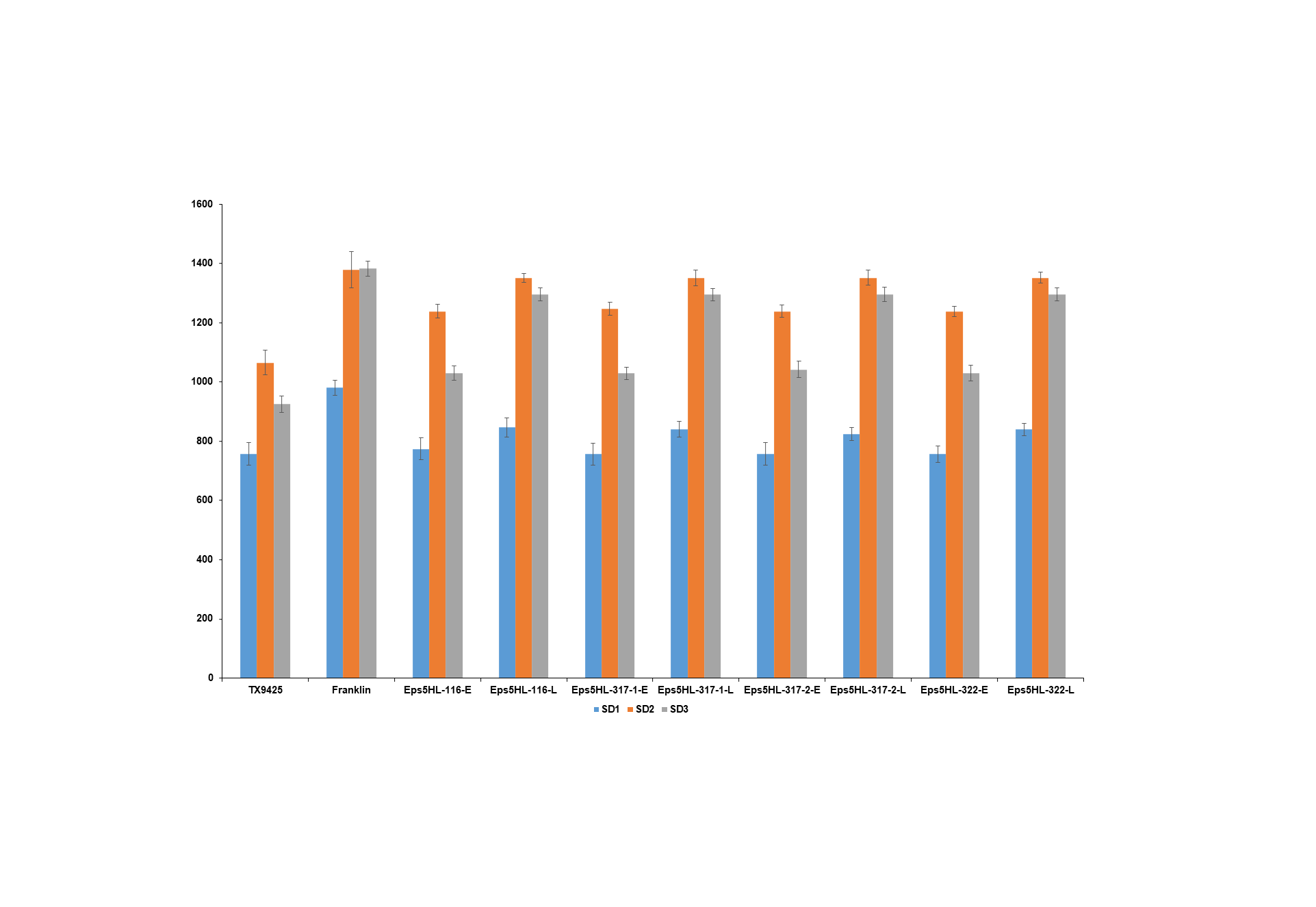

Supplement: S2 Fig — (TIF) [file pone.0200722.s002.TIF]

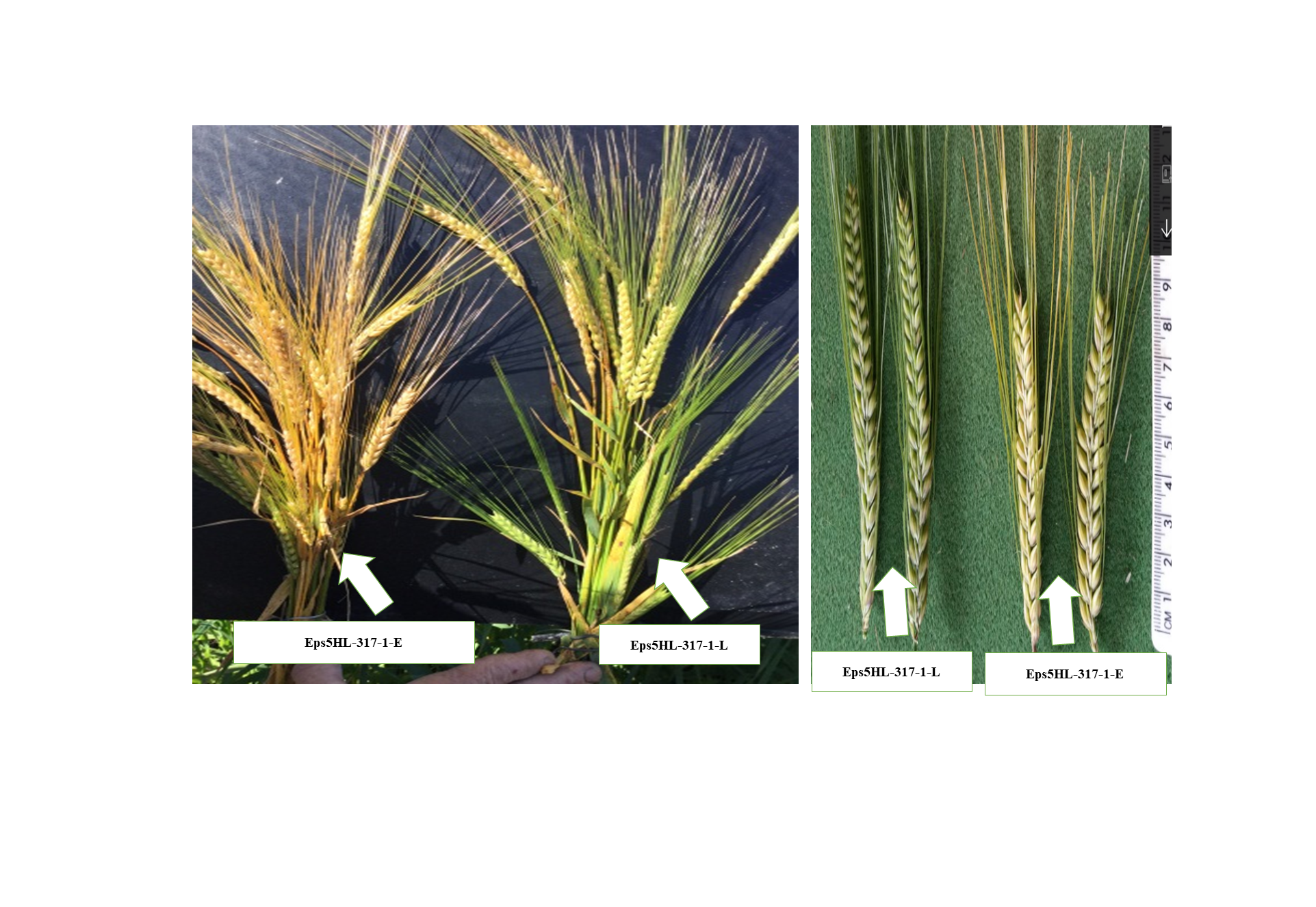

Supplement: S3 Fig — (TIF) [file pone.0200722.s003.TIF]
