# Supplementary material for: A regulator of early flowering in barley (Hordeum vulgare L.)
Source: PLoS One. 2018 Jul 17;13(7):e0200722. doi: 10.1371/journal.pone.0200722 (PMC6049932; doi:10.1371/journal.pone.0200722)
Supplement: S1 Table — (DOCX) [file pone.0200722.s004.docx]

**Table S1: Mean square values from the analysis of variance for all the traits studied for each pair of the NILs (early and late) and the parents (TX9425 and Franklin).**

| **S/variation** | **HD** | **GDD/TT** | **PH** | **SpkL** | **SpkN.** | **PedL** | **InterL** |
| --- | --- | --- | --- | --- | --- | --- | --- |
| **Eps5HL-116 pair** | 840.5** | 90291.25** | 0.067 | 26.94** | 35.26** | 1.159 | 0.63 |
| **SD** | 19522** | 401622.8** | 1123.43** | 11.05** | 335.3** | 420.6** | 39.6** |
| **Eps5HL-116 x SD** | 105.5** | 12505.76** | 0.096 | 0.039** | 6.12** | 64.103 | 11.627 |
| **Err** | 0.0023 | 0.0011 | 5.8 | 0.002 | 0.133 | 9.89 | 1.631 |
|  |  |  |  |  |  |  |  |
| **Eps5HL-317-1 pair** | 840.5** | 90291.25** | 305.05* | 27.30** | 34.68** | 30.86 | 22.819 |
| **SD** | 19522** | 401622.8** | 686.362** | 11.30** | 322.0** | 98.562* | 15.048 |
| **Eps5HL-317-1 x SD** | 105.5** | 12505.76** | 24.48 | 0.046** | 3.94** | 2.18 | 20.24 |
| **Err** | 0.0023 | 0.0011 | 22.74 | 0.004 | 0.18 | 19.6 | 3.75 |
| **Eps5HL-317-2 pair** | 840.5** | 90291.25** | 134.48* | 26.94** | 38.2** | 19.22 | 0.831 |
| **SD** | 19522** | 401622.76** | 786.80** | 10.98** | 369.5** | 55.822 | 0.265 |
| **Eps5HL-317-2 x SD** | 105.5** | 12505.76** | 10.535 | 0.034** | 7.38** | 5.01 | 16.433 |
| **Err** | 0.0023 | 0.0011 | 8.8167 | 0.0022 | 0.25 | 15.492 | 9.156 |
| **Eps5HL-322 pair** | 840.5** | 90291.25** | 122.201** | 26.82** | 34.27** | 21.63 | 2.722 |
| **SD** | 19521** | 401622.8** | 1210.89** | 11.525** | 359.93** | 170.897* | 3.423 |
| **Eps5HL-322 x SD** | 105.5** | 12505.76** | 0.354 | 0.0705** | 5.713** | 9.953 | 21.180* |
| **Err** | 0.0023 | 0.0011 | 2.706 | 0.0016 | 0.172 | 24.992 | 2.841 |
| **TXFRAN** | 4867.55** | 495186.64** | 408.98** | 159.60** | 72** | 4.18 | 21.24 |
| **SD** | 16002** | 218800.35** | 4653.37** | 17.85** | 298.5** | 18.6 | 3 |
| **Par*SD** | 438.88** | 20723.39** | 478.96** | 2.73** | 3.5 | 19.22 | 0.831 |
| **Err** | 8.36667 | 1119.057 | 9.311 | 0.033 | 2.4 | 50.822 | 1.265 |

** Highly Significant at 0.01 and * Significant at 0.05 probability level.
