# Supplementary material for: A regulator of early flowering in barley (Hordeum vulgare L.)
Source: PLoS One. 2018 Jul 17;13(7):e0200722. doi: 10.1371/journal.pone.0200722 (PMC6049932; doi:10.1371/journal.pone.0200722)
Supplement: S2 Table — (DOCX) [file pone.0200722.s005.docx]

**Table S2: Mean square values from the analysis of variance for all the pasting properties studied for each pair of the NILs (early and late) and the parents (TX9425 and Franklin)**

| **Sources of variance** | **Peak Viscosity** | **Trough** | | **Break-down** | | **Final viscosity** | | **Setback** | **Time to PV** | **P/Temp** |
| --- | --- | --- | --- | --- | --- | --- | --- | --- | --- | --- |
| **Eps5HL- 116** | 1638.7 | 483.1 | | 342.3 | | 1903.9 | | 468.9 | 0.002 | 0.053 |
| **Eps5HL-317-1** | 896.1 | 1523.1 | | 83.1 | | 6890.6 | | 1934.4 | 0.004 | 2.862 |
| **Eps5HL-317-2** | 1031.3 | 1212.7 | | 7.3 | | 2366.1 | | 190.9 | 0.014 | 10.580 |
| **Eps5HL-322** | 168.0 | 84.5 | | 14.2 | | 1098.6 | | 1792.5 | 0.045 | 5.695 |
| **TX9425/Franklin** | 11630.6* | | 18021.9 | | 696.9 | | 31284.2 | 1817.1 | 0.018 | 28.170 |

** Highly Significant at 0.01 and * Significant at 0.05 probability level
